# Large Unilateral Pleural Effusion with Pacemaker-associated Post-cardiac Injury Syndrome

**DOI:** 10.7759/cureus.2946

**Published:** 2018-07-08

**Authors:** Sundeep Kumar, Abed Madanieh, Hiren Patel, Ruthvik Srinivasa Murthy, Jose M Goyos, Mark R Milunski

**Affiliations:** 1 Internal Medicine, University of Central Florida College of Medicine, Orlando, USA; 2 Pulmonary and Critical Care, Orlando Veterans Affairs Medical Center, Orlando, USA; 3 Cardiology, Orlando Veterans Affairs Medical Center, Orlando, USA

**Keywords:** pleural effusion, post cardiac injury syndrome, pericarditis, permanent pacemaker, pericardial effusion, pacemaker associated post cardiac injury syndrome

## Abstract

Post-cardiac injury syndrome (PCIS) as a delayed complication of permanent pacemaker implantation has rarely been reported in the literature. A 67-year-old man who recently underwent a dual chamber permanent pacemaker implantation came to the hospital for increasing dyspnea and chest discomfort. A diagnosis of pericarditis was made, and the patient was discharged on ibuprofen therapy. He presented to our facility a month later with worsening dyspnea and chest discomfort despite recommended therapy. A computerized tomography (CT) scan of the chest revealed a large right-sided pleural effusion, requiring chest tube placement and drainage. A pleural fluid analysis revealed exudative effusion with elevated pH. The pleural fluid analysis was negative for infectious etiology. A perforation of the atrial wall was considered given the proximity of the atrial pacer lead and overlying pericardial effusion. However, no conclusive evidence of cardiac chamber perforation was found on echocardiogram or CT scan. A pacemaker interrogation was normal. A repeat CT scan showed the resolution of pleural effusion, and the chest tube was discontinued. A possible explanation for the absence of predominant pericardial findings may be the previous use of non-steroidal anti-inflammatory therapy.

## Introduction

Post-cardiac injury syndrome (PCIS) is a heterogeneous group of disorders occurring in response to a variety of cardiac injuries, including cardiac surgery, blunt chest wall trauma, and coronary and endovascular interventions, among others. PCIS can occur following cardiac device placement, and only a limited number of cases have been reported in the literature. We present a case of delayed PCIS after permanent pacemaker insertion presenting as a large unilateral pleural effusion. A cardiac perforation was also considered in the differential diagnosis because of the proximity of the pacemaker leads to the atrial wall and the associated pleuro-pericardial effusion, causing diagnostic and therapeutic challenges.

## Case presentation

**Figure 1:**
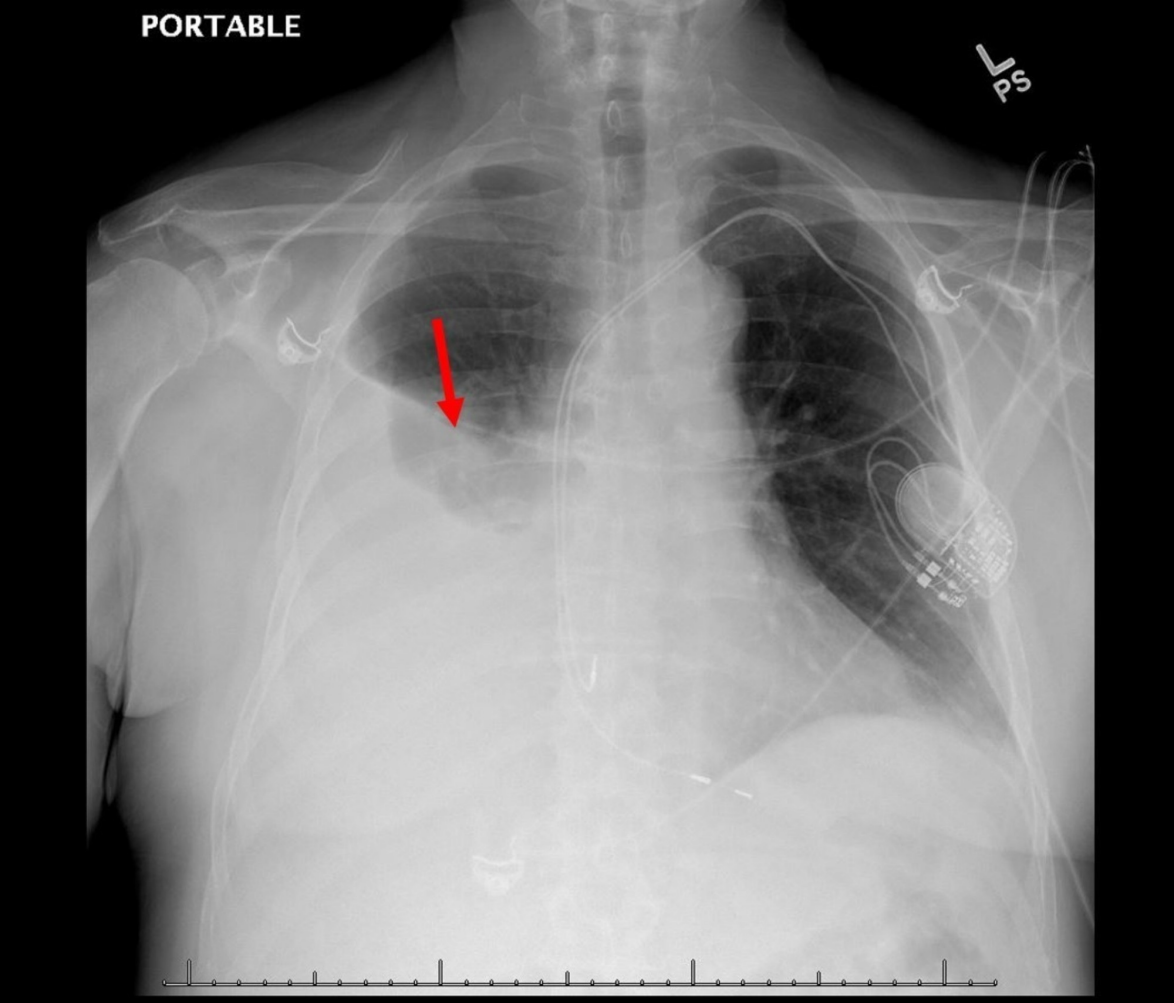
Chest radiograph (anterioposterior view) revealing large right-sided pleural effusion (red arrow)

**Figure 2:**
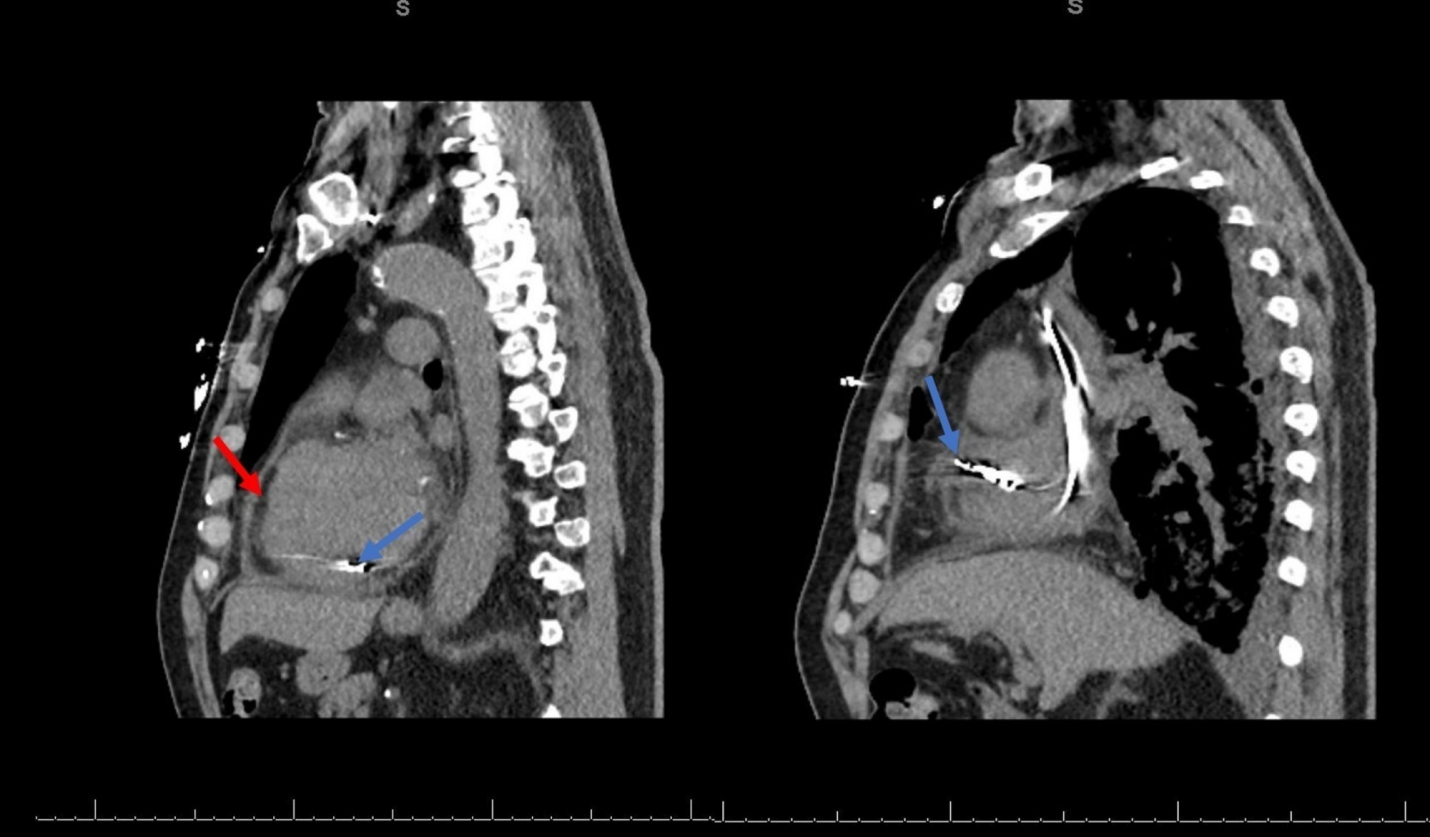
Computerized tomography scan of chest showing atrial pacemaker leads (blue arrows) in proximity to the atrial wall and overlying small-moderate pericardial effusion (red arrow)

A 67-year-old non-smoker man with a past medical history of non-obstructive coronary artery disease, hyperlipidemia, essential hypertension, paroxysmal atrial fibrillation, and subclinical hypothyroidism and no prior history of autoimmune disease in the family underwent permanent dual chamber pacemaker implantation for sinus node dysfunction. The patient was discharged home without any immediate procedural complications but returned to the hospital two weeks later with increasing dyspnea and chest discomfort. He had extensive testing, including workup for ischemic heart disease. A left heart catheterization was done, revealing non-obstructive coronary artery disease. A transthoracic echocardiogram revealed a small pericardial effusion without any other echocardiographic abnormalities. He was discharged home on ibuprofen with a diagnosis of pericarditis. He was admitted to our facility a month later with worsening dyspnea and non-productive cough. He denied fever, chills, or chest pain at presentation. The physical examination was consistent with decreased breath sounds in the right middle and lower lung fields. Chest radiograph (Figure 1) and computerized tomography (CT) of the chest (Figure 2) revealed a large right-sided pleural effusion and a small-moderate pericardial effusion. Pertinent laboratory workup showed no leukocytosis, hemoglobin of 11 g/dl, and a supratherapeutic international normalized ratio (INR) of 3.5 secondary to warfarin use. He received empiric antibiotics for a possible pulmonary infectious process. He received fresh frozen plasma and vitamin K to reverse the coagulopathy and underwent pleural fluid drainage with chest tube placement. A total of three liters of serosanguinous fluid was removed. The pleural fluid analysis was consistent with an exudative effusion using Light’s criteria with a pleural fluid/serum protein ratio of 0.625, a pleural fluid/serum lactate dehydrogenase (LDH) ratio of 1.526, and a pleural fluid LDH > 2/3 upper limit of normal plasma levels. Pleural fluid pH was elevated to 8.6 (normal: 7.60-7.65). Pleural fluid microbiology, including bacterial, fungal, and acid-fast bacilli cultures, were negative. Relevant laboratory and microbiologic data are shown in Table 1. A repeat CT scan of the chest following chest tube placement showed marked interval improvement in pleural effusion, and the chest tube was removed. On the chest CT scan, there was a concern for possible right atrial pacemaker lead causing the perforation of the right atrial wall due to its close proximity to the atrial wall and the associated pericardial effusion (Figure 2). However, no conclusive evidence of cardiac perforation was identified on repeat imaging and the patient remained clinically stable during the hospitalization. A pacemaker interrogation was normal. He had a repeat transthoracic echocardiogram three weeks later, which didn’t reveal any evidence of atrial perforation, including no pericardial effusion. A follow-up chest radiograph demonstrated near-complete resolution of the pleural effusion. This case represents an unusual presentation of PCIS where symptoms were predominantly pulmonary, including a large unilateral pleural effusion.

**Table 1:** Laboratory and microbiology data

Laboratory and Microbiology Data	
Pleural fluid lactate dehydrogenase	435 U/L
Pleural fluid glucose	108 mg/dl
Pleural fluid protein	4.0 g/dl
Serum lactate dehydrogenase	285 U/L
Serum protein	6.4 g/dl
Fungal smear (pleural fluid)	Negative
Acid-fast bacilli culture (pleural fluid)	Negative
Bacterial culture (pleural fluid)	Negative
Color (pleural fluid)	bloody
Total leukocytes (pleural fluid)	1625 #/cmm
Segmented cells (pleural fluid)	14%
Lymphocytes (pleural fluid)	72%
Erythrocytes (pleural fluid)	33750/cmm

## Discussion

Post-cardiac injury syndrome (PCIS) is an inflammatory process involving the pleura and pericardium in response to cardiac injury. PCIS can occur in response to endocardial, myocardial, or epicardial injury and has been described after percutaneous coronary interventions. PCIS is infrequently reported in association with pacemaker device insertion or radiofrequency ablation with only a few case reports in the literature [1-3]. PCIS typically presents with concomitant pleural and pericardial involvement in the form of effusions in the majority of the cases reported. There are no reported cases of solitary or predominant pulmonary involvement. Pathognomonic features include dyspnea, fever, pleuropericardial pain, and pericardial involvement with or without effusion, pulmonary involvement in the form of effusions or parenchymal disease, and elevated inflammatory markers. The exact mechanism of pacemaker-induced PCIS is poorly understood but may involve localized microtrauma at the site of pacemaker lead insertion.

The spectrum of PCIS ranges from pericarditis with pericardial effusion to massive pleuropericardial effusions and cardiac tamponade. Pericardial effusion with or without the features of tamponade was found in the majority of cases [4]. PCIS may resemble delayed cardiac perforation from cardiac device placement [5]. Pericardial effusion resulting from pacemaker-induced perforation is common with concomitant steroid use, temporary pacemaker placement, increasing age, low body mass index (BMI), and helical screw lead use [6]. Epicardial pacemaker implantation and active fixation atrial leads are associated with an increased risk of PCIS [7]. The increased incidence of PCIS and cardiac perforation with atrial leads are likely related to the thin atrial wall. Dyspnea, fever, and elevated inflammatory markers should raise the suspicion for PCIS. In our patient, cardiac perforation was also considered. However, no objective evidence of perforation was demonstrated on CT scan, transthoracic echocardiography, or pacemaker interrogation.

It is important to differentiate PCIS from pacemaker lead perforation to prevent unnecessary diagnostic and therapeutic interventions. Lead perforation may cause a change in pacing and sensing parameters on pacemaker interrogation. However, PCIS is less likely to cause such changes. Concomitant anticoagulation, female gender, and steroid use increases the risk of lead perforation but don’t affect the risk of developing PCIS [6]. Instead, current steroid use may theoretically prevent or decrease the severity of PCIS. Right-sided heart perforation can mimic PCIS and go unrecognized because of the low-pressure system [8].

Pleuropulmonary manifestations of PCIS have been reported but rarely with permanent pacemaker insertion. The pleural fluid analysis reported exudative effusions in the majority of cases. It may be difficult to differentiate the exudative effusion seen with PCIS from an infectious cause especially early in the course of the illness. A high pleural fluid pH may favor the diagnosis of PCIS over infectious causes, as seen in our patient [9]. A large registry of pleural fluid analysis in patients with PCIS reported that all the effusions were exudative, occupied less than one-third of the hemithorax and was hemorrhagic in most cases [10]. Therefore, the pleural fluid analysis may provide valuable diagnostic information.

Investigations for pacemaker-associated PCIS should include device interrogation, chest radiography, transthoracic or transesophageal echocardiography, computerized tomography (CT) of the chest, and laboratory studies, including complete blood counts, and inflammatory markers, including erythrocyte sedimentation rate (ESR). Some investigators have suggested a role of anti-heart antibodies in the diagnosis; however, their clinical significance remains unclear [11]. A CT scan of the chest is the best way to detect pleuropericardial involvement in PCIS and can help in identifying cardiac perforations. Elevated ESR, although a nonspecific finding, is present in the majority of PCIS cases.

## Conclusions

This case is unusual because of the development of a large pleural effusion despite being treated with recommended therapy and the absence of predominant pericardial symptoms. A possible explanation for the absence of predominant pericardial findings may be the previous use of non-steroidal anti-inflammatory medication. Clinical, radiographic, and laboratory features are helpful in differentiating between PCIS and lead perforation. A pleural fluid analysis showing a high pH is helpful in the diagnosis of PCIS. A chest CT scan may be helpful in cases with an unclear diagnosis, but this may fail to provide a clear diagnosis in some cases. A delayed cardiac perforation from pacemaker leads is a life-threatening condition that can mimic PCIS and should always be considered. A high pleural fluid pH helps differentiate an effusion due to PCIS from one caused by a pulmonary infectious process that is usually associated with low pH.
